# Supplementary material for: Evidencing New Roles for the Glycosyl-Transferase Cps1 in the Phytopathogenic Fungus Botrytis cinerea
Source: J Fungi (Basel). 2022 Aug 24;8(9):899. doi: 10.3390/jof8090899 (PMC9500679; doi:10.3390/jof8090899)
Supplement: Supplementary file 1 [file jof-08-00899-s001.zip › Supp finale/text.pdf]

Figure S1. Evolution of Cps1-like proteins. A ML phylogeny based on 292 amino acid alignment positions of 78 sequences was constructed with PhyML. F1 (blue branches) and F2 (green branches) correspond respectively to the *Leotiomyceta* Cps1 and Cps2 subfamilies. F0 (black branches) gathers other proteins. Bootstraps ≥ 80 are shown. The gene names of the characterized Cps1-like protein encoding genes (Figure 1) are added in bold beside sequence names. The abbreviations used in sequence names are listed in Table S2.

Figure S2. Fixation of the Cps1 subfamily genes in the *Leotiomyceta* genomes. The diagrammatic representation of the fungal tree of life is adapted from King et al., 2017 and the NCBI taxonomy. Each fungal taxonomic class or subphylum is shown with brackets highlighting the number of genomes analysed. Blue shading in pie charts indicate the proportion of species with at least one Cps1-like encoding gene belonging to the F0, F1 (Cps1 subfamily) or F2 (Cps2 subfamily) groups described in Figure S1. The D letter indicated beside the *Leotiomyceta* node indicates the event of duplication at the origin of the Cps1 and Cps2 subfamilies. The category undefined correspond to Cps1-like proteins not classified with the phylogenetic tree of which they were removed as they provoked long branches. The crosses indicate the independent losses of Cps1-like encoding genes that have occurred in ascomycetes.

Figure S3. DNA cassettes and molecular characterizations of the genetically modified strains. (**A**) Fungalbraid assemblage scheme of the *Bccps1* 5′ and 3′ flanking regions (FR) to the FB012 biobrick containing the hygromycin-resistance gene (*Hph*) under the p*TrpC* promoter. The barcodes used to drive the assemblage of the deletion cassette are indicated in capital letters. (B) Scheme of the gene replacement strategy used to create the Δ*Bccps1* mutant strain. Dashed lines: expected double recombination between the *Bccps1* 5′ and 3′ FR present in the targeted locus (top) and in the *A. tumefaciens* T-DNA carrying the deletion cassette. LB, RB: Left and right borders. Red bar: DNA probe used in the Southern blotting experiment. (C) Scheme of the complementation DNA cassette used to create the Δ*Bccps1-C* strain. Neo: neomycin-resistance gene. P: promoter. T: terminator. CDS: coding sequence. (D) Southern blot analysis of the parental (B05.10) and deletion (Δ*Bccps1*) strain. Genomic DNA fragments produced by EcoRI digestion were separated by gel electrophoresis, transferred to a nylon membrane and hybridized with the probe shown in (B). (E) PCR diagnostic of the deletion and complemented strains. The primers used are indicated at the bottom of the gel images and as arrows in B and C.

Figure S4. BcCps1-GFP fusion proteins and localization by confocal microscopy. (A) Three BcCps1-GFP encoding DNA cassettes were cloned under the control of the strong constitutive PoliC promoter and introduced in *B. cinerea* by ATMT transformation. The sequence of the 3 expected fusion proteins is shown with the 3 eGFP insertions sites (green). The predicted TM domains and the catalytic domains are coloured in brown and blue, respectively. In red, putative NRD/Nardilysin, Furin and Yeast-Kexin-2 protease cleavage sites. The linkers added between the GFP and BcCps1 sequences are indicated in brackets. (B) Confocal microscopy images showing the vacuolar localisation of BcCps1 fused to eGFP at the C-terminus.

Figure S5. Conidia germination. Conidia of the parental (B05.10) and mutant (Δ*Bccps1*) strains were inoculated in liquid rich medium in a micro-plate. At 4.5 and 9 hours post-inoculation, the conidia

producing a germ tube were counted under the microscope. Means and standard deviations were calculated from 3 independent experiments (n = 100).

Figure S6. Sensitivity to stresses and cell wall contents in chitin and lichenan. (**A**) Mycelial growth of the parental (B05.10) and mutant (Δ*Bccps1*) strains in the presence of 0.6 M KCl (1.2 osmol), 0.02% SDS or menadione (56 μg/mL). (**B**) Following digestion of cell wall extracts of the parental and mutant strains by lyzing enzymes or a lichenase (targeting β-1,3-1,4-glucan), free N-acetyl-glucosamine (left) and free sugars were quantified spectrophotometrically (1 OD^{585} = 8 μg glucosamine & 0.1 OD^{414} = 1 μg glucose). Means and standard deviations were calculated from 3 independent experiments (n = 9). T-test bilateral; $p<0.01$.

Figure S8. Enzymatic activities in the secretome of the complemented strain. Supernatants of rich medium liquid cultures of the Δ*Bccps1-C* strain were used to measure enzymatic activities. The results are shown as percentages of the parental strain values. Means and standard deviations were calculated from 4 independent experiments (n = 12). Prot; proteases. Xyl; xylanases. Lac; laccases. PMEs; pectin methyl esterases. Amy; amylases.
